# Nonallergic Rhinitis, With a Focus on Vasomotor Rhinitis *Clinical Importance, Differential Diagnosis, and Effective Treatment Recommendations*

**DOI:** 10.1097/WAO.0b013e318196ca1e

**Published:** 2009-03-15

**Authors:** Mark D Scarupa, Michael A Kaliner

**Affiliations:** 1 Institute for Asthma & Allergy, 6515 Hillmead Road, Bethesda, MD 20817

**Keywords:** nonallergic rhinitis, vasomotor rhinitis, mixed rhinitis, allergic rhinitis

## Abstract

The term "rhinitis" denotes nasal inflammation causing a combination of rhinorrhea, sneezing, congestion, nasal itch, and/or postnasal drainage. Allergic rhinitis is the most prevalent and most frequently recognized form of rhinitis. However, nonallergic rhinitis (NAR) is also very common, affecting millions of people. By contrast, NAR is less well understood and less often diagnosed. Nonallergic rhinitis includes a heterogeneous group of conditions, involving various triggers and distinct pathophysiologies. Nonallergic vasomo-tor rhinitis is the most common form of NAR and will be the primary focus of this review. Understanding and recognizing the presence of NAR in a patient is essential for the correct selection of medications and for successful treatment outcomes.

## 

Nonallergic rhinitis (NAR) is not a single disease with 1 underlying mechanism but is instead a collection of multiple distinct conditions that cause similar nasal symptoms. Nonallergic rhinitis is at times almost indistinguishable from allergic rhinitis (AR), although typically nasal and palatal itch, sneezing, and conjunctival irritation are less prominent. Non-allergic rhinitis can and frequently does exist simultaneously with AR, a condition known as "mixed rhinitis." The most clinically prevalent form of NAR is vasomotor or idiopathic rhinitis, characterized by sporadic or persistent perennial nasal symptoms that are triggered by environmental conditions, such as strong smells; cold air; changes in temperature, humidity, and barometric pressure; strong emotions; ingesting alcoholic beverages; and changes in hormone levels. These triggers do not involve immunoglobulin E cross-linking or histamine release.

## Epidemiology and impact

The incidence of NAR varies from study to study. Almost all publications on NAR are found in North American and European literature. Thus, it is unclear whether the incidence or the age and sex distribution applies to populations not yet studied elsewhere in the world. In 1 survey of US medical practices, the classification of patients with rhinitis was 43% AR, 23% NAR, and 34% mixed rhinitis (rhinitis with both AR and NAR features) [1]. These data suggest that at least 57% of rhinitis patients have some contribution from NAR causing their rhinitis symptoms. Similar European studies have found that approximately 1 in 4 patients complaining of nasal symptoms has pure NAR [2]. Recent estimates suggest that 50 million Europeans have NAR, with a total prevalence of greater than 200 million worldwide [3]. In the United States, there are approximately 60 million patients with AR and 30 million with nonallergic vasomotor rhinitis (VMR).

Nonallergic rhinitis tends to be adult onset, with the typical age of presentation between 30 and 60 years [4]. Once symptoms begin, they frequently last a lifetime. If NAR is present in pediatric populations, it is more likely to be anatomic in nature and to be caused by either adenoid or turbinate hypertrophy, leading to persistent nasal obstruction. In adults, most studies report a clear female predominance, with estimates ranging from 58% to 71% of those affected being female. In a Danish study classifying a population of both adults and adolescents, female predominance held true with approximately double the prevalence of NAR in women [2].

The financial impact of NAR has not been studied directly, but numerous studies have looked at the direct and the indirect costs of AR. It is likely that because most studies indicate that at least 1 in 4 patients with nasal symptoms have pure NAR, the rough cost of the condition is approximately one third of AR. Direct and indirect US medical expenditures for AR are in excess of 2.7 billion dollars (1995 dollars) [5]. When lost productivity due to drowsiness, cognitive/motor impairment, and missed school and work is considered, the cost estimate increases to $6 billion [6]. Thus, although no reports of the costs of NAR have been reported, it is likely that this disease costs at least US$2 to 3 billion per year.

## NAR: Diagnosis and classification

Nonallergic rhinitis denotes a group of heterogeneous syndromes with distinct underlying pathophysiologies. Historically, NAR variants have been divided into 2 groups based on nasal cytology: NAR with eosinophilia syndrome (NARES) and non-NARES. However, in this era, nasal cytology is rarely performed in clinical practice. Thus, it is logical today to classify NAR based solely on symptoms and triggers. Furthermore, when considering the diagnosis of NAR, the concomitant presence of AR and/or chronic rhino-sinusitis needs to be considered. Most patients with chronic nasal symptoms appear at the physician's office assuming that they have AR. As part of the diagnostic steps used to confirm the diagnosis, most patients undergo specific environmental allergy testing either by skin test or by radioallergosorbent test. In the presence of negative allergy skin tests and a history of rhinitis symptoms, most patients will have some form of NAR or rhinosinusitis.

Nonallergic rhinitis can be classified into 9 subtypes (Table 1): drug-induced rhinitis, gustatory rhinitis (rhinorrhea associated with eating), hormonal-induced rhinitis, infectious rhinitis, NARES, occupational rhinitis, senile rhinitis, atrophic rhinitis, and VMR (modified from Settipane and Charnock[4]). Rhinitis of pregnancy is an extremely common condition effecting up to 20% to 30% of pregnancies, especially notable during the last trimester [7]. It typically resolves spontaneously within 2 weeks of delivery. As 1 clue to specific causes, Ellegard et al[8] have shown that woman with rhinitis of pregnancy have elevated serum placental growth hormone levels when compared with pregnant women without rhinitis. However, it is usually assumed that the rhinitis of pregnancy reflects the mucosal engorgement found in the last trimester as a consequence of progesterone stimulation. Thus, the nasal mucosa also becomes engorged and congestion ensues.

**Table 1:** Specific Syndromes Classified as NAR

Drug-induced rhinitis, including rhinitis medicamentosa
Gustatory rhinitis
Hormonal-induced rhinitis, including the rhinitis of pregnancy
Infectious rhinitis
NARES
Occupational rhinitis
Senile rhinitis
Atrophic rhinitis
Vasomotor or idiopathic rhinitis

Rhinitis medicamentosa or medication-induced rhinitis is another common NAR variant. The most common cause of rhinitis medicamentosa is overuse of the topical nasal decongestants oxymetazoline or phenylephrine. When used briefly (less than 3-5 days consecutively), these medications provide significant relief of nasal congestion. However, with chronic use, rebound nasal congestion can occur and can be quite severe. The exact mechanism is poorly understood, but theories involving recurrent nasal tissue hypoxia and negative neural feedback with chronic α-2 receptor agonism exist [9]. Rhinitis medicamentosa is treated with topical nasal corticosteroids (NCCSs) and/or oral corticosteroids, and progressive withdrawal of the topical decongestant sprays over a 3- to 7-day period.

More broadly, other medications can cause chronic nasal symptoms through a host of different mechanisms. Antihypertensive medications including β blockers, reserpine, calcium channel blockers, and methyldopa frequently cause nasal congestion [1]. Aspirin and nonsteroidal anti-inflammatory drugs also may contribute to congestion especially in patients with a history of nasal polyposis. Oral contraceptive pills also can cause congestion in some women. Eye drops can cause rhinitis after they pass the nasolacrimal duct into the nose.

Nonallergic rhinitis also is also found with other underlying medical conditions, the full range of which is beyond the scope of this article (Table 2). For example, nasal congestion can be seen in disparate diseases such as hypothyroidism and chronic fatigue syndrome. Baraniuk et al[10] found that 46% of patients with chronic fatigue syndrome have NAR and that 76% of patients have some nasal complaints. Gastroesophageal reflux disease or laryngeal-pharyngeal reflux can lead to chronic postnasal drip and other throat symptoms and, in severe cases, can also cause nasal congestion.

**Table 2:** Medical Conditions Associated With or Presenting Similarly to NAR

Metabolic
Acromegaly
Pregnancy
Hypothyroidism
Autoimmune
Sjogren syndrome
Systemic lupus erythematosus
Relapsing polychondritis
Churg-Straus syndrome
Wegner granulomatosis
Other
Cystic fibrosis
Kartagener syndrome
Sarcoidosis
Immunodeficiency

Adapted from Settipane and Settipane [1].

Anatomic anomalies can contribute to NAR. Both adenoid hypertrophy and turbinate hypertrophy can cause symptoms of chronic nasal congestion with little relief from medications. Surgical intervention can be curative. Quite dissimilar to the anatomic hypertrophies, senile rhinitis is most common in the elderly and can lead to persistent rhinorrhea, worsened by eating or environmental irritants. Atrophic rhinitis is usually seen in patients who have had overzealous surgeries, with too much mucus-secreting tissues removed. Cerebral spinal fluid leak in patients with a history of craniofacial trauma or past facial/sinus surgeries must be considered when evaluating persistent rhinorrhea.

### Nonallergic VMR

The most frequent form of NAR observed clinically is nonallergic VMR or idiopathic rhinitis, characterized by sporadic or persistent nasal symptoms that are triggered by environmental conditions, such as strong smells; exposure to cold air; changes in temperature, humidity, and barometric pressure; strong emotions; ingesting alcoholic beverages; and changes in hormone levels. The diagnosis of VMR is primarily made by clinical history. If a patient has appropriate nasal symptoms (usually rhinorrhea, congestion, postnasal drip, headaches, throat clearing, and coughing) triggered by 1 or more environmental irritants, then VMR is present. Concomitant ocular symptoms tend to be minimal, and the symptoms of nasal and palatal itch as well as sneezing spells are not common. Unlike AR, VMR is usually of adult onset and not worsened by exposure to classic allergens such pollen, house dust mite, dog, or cat. A validated questionnaire has been created to help identify NAR patients [11]. Because VMR may be caused by shifts in temperature and humidity, patients may experience seasonal symptoms during spring and fall. Thus, seasonal VMR can easily be confused with Seasonal Allergic Rhinitis (SAR) [12].

The diagnosis of VMR is based solely on the patient's history of symptoms and their triggers, whereas the diagnosis of AR requires an appropriate history and confirmatory allergy testing, either positive prick skin tests or radioallergosorbent tests. These diseases are not mutually exclusive, and nearly 60% of AR patients have a component of VMR participating in triggering symptoms. In 1 survey of patients with chronic rhinosinusitis, AR was the underlying cause in 65%, whereas VMR was coexistent in 25% [13]. Thus, there is an extensive overlap between the 3 most common chronic nasal diseases: AR, VMR, and chronic rhinosinusitis.

The exact underlying mechanisms causing VMR are not well understood. There is evidence that capsaicin-sensitive nociceptors in the nasal mucosa may play a role [14-16]. Other studies have shown that in patients with cold-air-induced NAR, inhalation of cold air into 1 nostril causes contralateral symptoms that can be blocked by pretreating the challenged nostril with lidocaine or repetitively treating the nose with capsaicin before challenge [17,18]. Further evidence of a neurologic mechanism driving NAR is a small study suggesting that endoscopic vidian neurectomy reduces both rhinorrhea and congestion in VMR patients [19]. Some studies have also demonstrated that topical nasal capsaicin treatment of VMR patients can induce prolonged symptom relief [15]. By contrast, there is some data that mast cells can be activated in cold-air-induced rhinitis by breathing cold dry air, which caused in vivo histamine release in cold-air-induced rhinitis but not in other forms of VMR [20].

The epidemiologic predominance of females experiencing VMR suggests that female hormones might play some role, but there is no research explaining this possibility.

## Treatment

**Figure 1:**
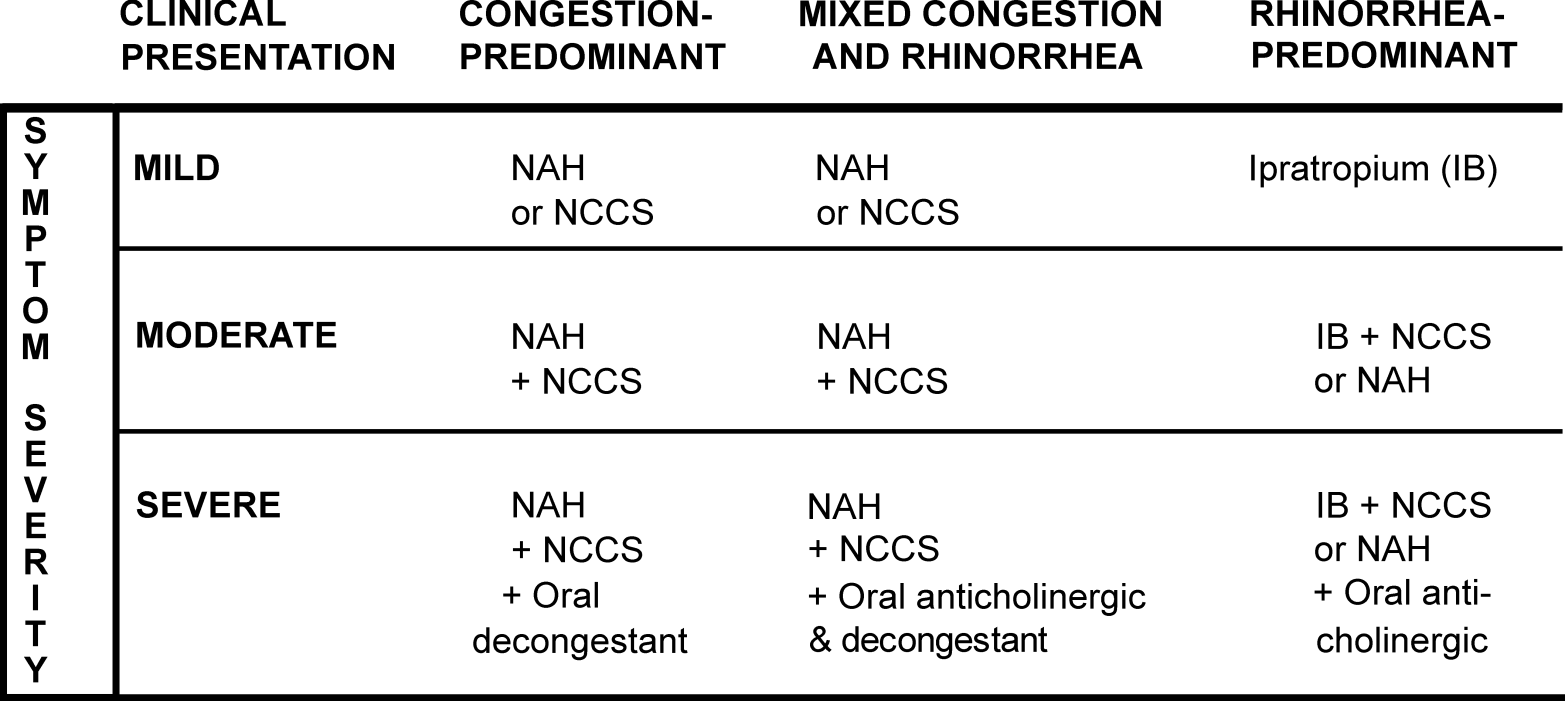
**Algorithm for the treatment of nonallergic VMR**. Once a patient is categorized as VMR, the predominant symptom complex determines initial treatments based on symptom severity. Initial treatments for mildly affected patients use single entities, but patients with more severe disease who have failed monotherapy should be tried on combination therapies. Most patients will ultimately respond to the use of combinations of nasal sprays plus an oral medication. Once under control, stepping the therapy down to the lowest effective dose of mediations is suggested.

Although each form of NAR should be treated individually, VMR is the most well-studied and clinically important form of NAR and the only type of NAR for which clinical studies have led to approved treatments. In the following discussion, treatment of NAR will focus on VMR, but some mention will be made of other forms of NAR where appropriate. The medications used for treating VMR have been studied less extensively than those for AR, but there are still multiple therapeutic options available (Figure 1). In Figure 1, the algorithm is based on separation of VMR into 3 clinical presentations: congestion predominant, rhinorrhea predominant, and mixed form of VMR where patients experience both rhinorrhea and congestion.

### Nasal corticosteroids

Nasal corticosteroids treat inflammatory conditions regardless of etiology. There is substantial evidence that corticosteroids benefit AR, some forms of NAR including VMR, and chronic rhinosinusitis. In a study of 983 patients with NARES and non-NARES, fluticasone propionate (FP) at both 200 and 400 μg significantly improved total nasal symptoms scores when compared with placebo, and no difference was noted between the 2 concentrations [21]. In the United States, of all the NCCSs approved by the Food and Drug Authority (FDA) available today, only FP is approved for the treatment of both AR and NAR.

Although none of the other current NCCS has received US FDA approval for use in NAR, there is some supportive data for the efficacy of intranasal budesonide and mometasone in some patients with perennial rhinitis [22,23]. There is also 1 published study demonstrating that there was no benefit from FP in NAR. In that study, NAR patients receiving 200 μg of daily FP showed a reduction in inflammatory mediators but no improvement in symptoms as compared with placebo [14]. By contrast, clinical experience suggests that all NCCSs have some effectiveness in treating VMR.

In VMR, the scent of fluticasone is sometimes a negative feature in patients for whom scent is a trigger. However, as a class, NCCS treats the broadest spectrum of NAR symptoms and seems to have at least some degree of efficacy in all NAR variants, including VMR. Thus, for the treatment of NAR, NCCSs are considered a first-line therapy.

### Antihistamines

It is quite likely that all NAR patients have tried oral antihistamines, either in the form of over-the-counter medications or as prescribed by physicians who assume that the symptoms are caused by allergies. Histamine release has not been seen in NAR and is specifically not seen in VMR other than cold-air-induced rhinitis [20]. Thus, the use of oral antihistamines makes little sense, and these medications have rarely been studied in VMR. A 1982 study does show that first-generation antihistamines can improve VMR symptoms when combined with a decongestant [24].

It is predictable that first-generation antihistamines might reduce rhinorrhea through anticholinergic actions, whereas second-generation nonsedating antihistamines have minimal anticholinergic activity. Typically, second-generation oral antihistamines are of no benefit in NAR. Oral antihistamines are generally ineffective in reducing congestion in AR and thus would not be expected to work in NAR either. The combination of an antihistamine and a decongestant might help reduce the congestion seen in VMR, but no such indication has been approved by the US FDA. Clinical experience suggests that antihistamine/decongestant combinations are somewhat effective in VMR.

By contrast, intranasal antihistamines are very effective in treating AR (both azelastine and olopatadine are approved for treating SAR). Azelastine is also approved by the FDA for treatment of nonallergic VMR. Although azelastine is primarily an antihistamine, it is unlikely that its efficacy in VMR is due to histamine receptor blockade. Instead, it is probably azelastine's actions as an anti-inflammatory and neuroinflammatory blocker that makes this medication useful in treating VMR or NAR. Azelastine has been shown to deplete inflammatory neuropeptides in the nasal mucosa; to reduce levels of proinflammatory cytokines, leukotrienes, and cell adhesion molecules; and to inhibit mast cell degranulation [25].

In 2 multicenter, randomized, double-blind, placebo-controlled, parallel-group clinical trials, azelastine showed considerable efficacy in the treatment of each of the symptoms of VMR or NAR, including congestion [26]. Treatment over 21 days caused a significant reduction in the total VMR symptom score from baseline when compared with placebo (*P *= 0.002), and every nasal symptom was effectively reduced. Symptom improvement was rapid with most patients experiencing relief within 1 week. There were no serious adverse events, although a bitter taste was experienced by some in the azelastine group. In studies of AR, onset of effect with nasal azelastine is seen in 15 to 30 minutes [25].

A meta-analysis has suggested that NCCSs are slightly more effective than azelastine in the treatment of AR, but no such analyses exist comparing these products in treating NAR [27]. When NCCS and azelastine were combined in the treatment of AR, the effects of the combination were additive. In a randomized double-blind trial comparing FP alone versus azelastine alone versus the two in combination for the treatment of AR, the combination produced a further 40% reduction in total nasal symptom scores as compared with either FP or azelastine alone. The combination of FP and azelastine reduced congestion by 48% compared with the individual components [28]. The combination has yet to be studied in VMR or NAR, but extensive clinical experience suggests that this combination is highly effective in VMR as well. On the basis of both published clinical studies and extensive clinical experience, the use of azelastine (and possibly olopatadine) alone and in combination with NCCS is a preferred first-line treatment of VMR/NAR as well as AR.

### Anticholinergics

Ipratropium bromide (IB) is a potent intranasal anti-cholinergic with utility in the treatment of rhinorrhea in AR and NAR. It has been studied in both adults and children. Ipratropium bromide specifically treats rhinorrhea and does little to improve congestion. Intranasal anticholinergics work best for rhinorrhea predominant NAR variants such as cold-air-induced rhinitis (skier's nose)[29] and gustatory and senile rhinitis [30]. In 28 patients with cold-air-induced rhinitis, IB reduced the symptoms and the number of tissues required during and after cold exposure (*P *= 0.0007 and 0.0023, respectively) [31]. In children with perennial AR or NAR, the effect of IB was superior to placebo and equivocal to beclomethasone dipropionate (BD) for the treatment of both rhinorrhea and congestion. However, IB was less effective than BD for controlling sneezing [32].

Similar to the nasal antihistamines, there seems to be an additive effect when IB is used in conjunction with NCCSs [33]. In a study comparing beclomethasone versus IB versus the two combined, the combination group had better symptom control of rhinorrhea. Beclomethasone monotherapy was found to better treat sneezing and congestion than IB monotherapy. Both medications were very well tolerated.

Oral anticholinergics such as methscopolamine have not been studied in NAR but likely improve symptoms particularly in rhinorrhea predominant disease or in patients with significant postnasal drainage. Many first-generation antihistamines and decongestants also have strong anticholinergic properties. However, side effects such as dry mouth, sedation, and urinary hesitancy limit the usefulness of these drugs. Clinical experience suggests that oral methscopolamine combined with an oral first-generation antihistamine is helpful in treating patients with postnasal drip and that adding this combination to nasal IB, nasal antihistamines, or NCCS is useful.

### Decongestants

Both oral and topical decongestants effectively treat congestion regardless of cause; however, none have been studied for NAR. Oral pseudoephedrine is an effective decongestant and can be considered for chronic use. However, side effects such as neurogenic and cardiac stimulation, palpitations, and insomnia affect a significant number of patients. Furthermore, the medication is relatively contraindicated in patients with hypertension. Thus, pseudoephedrine must be used cautiously. Phenylephrine is also an oral decongestant. It has been studied far less than pseudoephedrine and is considered a generally less potent medication.

Topical decongestants such as oxymetazoline and phenylephrine are fast acting potent local decongestants. These medications cannot be used chronically because continual use for more than 3 to 10 days leads to rhinitis medicamentosa. For NAR patients with intermittent nasal congestion, a topical decongestant can be used for short-term relief of congestion.

### Other NAR Therapies

Although NAR effects many patients, very few medications have been adequately studied for the treatment of this condition. In patients who do not adequately respond to NCCSs, intranasal antihistamines, or IB, other agents can be considered. There are a few limited studies examining intranasal capsaicin in NAR. In theory, repetitive capsaicin application depletes certain neuroinflammatory chemicals. Van Rijswijk et al[18] did demonstrate decreased nasal symptoms in VMR patients treated with capsaicin. Similarly, botulinum toxin A injected into the inferior and middle turbinates of patients with NAR has been shown to decrease congestion, sneezing, rhinorrhea, and nasal itch [34]. In patients with congestion-predominant NAR and turbinate hypertrophy, surgical reduction of the inferior turbinates may be of some benefit [35]. Nasal washing with isotonic or hypertonic saline has a demonstrated benefit particularly in chronic rhinosinusitis and seems to benefit some NAR patients [36]. Antileukotrienes have not been studied in NAR, but there is at least some theoretical benefit in patients with aspirin sensitivity and/or nasal polyposis. One controlled trial has demonstrated some efficacy using acupuncture in NAR [37].

## Conclusions

Nonallergic rhinitis is an underrecognized and inadequately treated condition affecting many subjects. Diagnosis is dependent on a thorough history and exclusion of other underlying conditions, including AR and chronic rhinosinusitis. Nonallergic rhinitis tends to require chronic medical management, and use of topical NCCSs and nasal antihistamines, used alone or in combination, is very effective in most patients. This combination is also extremely effective in treating AR. Thus, recognizing that the combination of both NCCSs and nasal antihistamines effectively treat AR, VMR, and mixed rhinitis, this combination of medications seems to be a useful first-line treatment for the overwhelming majority of rhinitis patients.
